# Hemophagocytic lymphohistiocytosis secondary to visceral leishmaniasis in children: case report and systematic review

**DOI:** 10.3389/fped.2025.1561600

**Published:** 2025-04-11

**Authors:** Zhu Chen, Yi Gao, Chaoyong Zhang, Junwen Mao

**Affiliations:** ^1^ Department of Pediatric Cardiology, Chengdu Women's and Children's Central Hospital, School of Medicine, University of Electronic Science and Technology of China, Chengdu, China; ^2^ Department of Pediatrics, Public Health Clinical Center of Chengdu, Chengdu, China; ^3^ Department of Pediatrics, The First Affiliated Hospital, Zhejiang University School of Medicine, Hangzhou, China

**Keywords:** visceral leishmaniasis, hemophagocytic lymphohistiocytosis, children, amphotericin B, systematic review

## Abstract

**Background:**

Visceral leishmaniasis (VL) can lead to complications such as hemophagocytic lymphohistiocytosis (HLH) in children. The clinical features of VL overlap with that of HLH, and thus the diagnosis of VL-induced HLH can be challenging for clinicians.

**Methods:**

We describe two pediatric cases and systematically review all reported cases of pediatric VL-related HLH in literatures until May 2024.

**Results:**

The demographic characteristics, clinical manifestations, treatment and prognosis of our reported cases are presented. The systematic review included 29 articles with a total of 135 cases. More than half of the children (89/125, 71.2%) were under 3 years old, and 8.9% (*n* = 12/135) had specific epidemiological histories. The main clinical presentations were hypertriglyceridemia (34/45, 75.6%), hypofibrinogenemia (24/36, 66.7%), and hyperferritinemia (95/132, 72.0%). Bone marrow aspiration (BMA) analysis indicated positive evidence of *leishmania* infection in 84.7% (83/98) of cases, while 37.8% (14/37) of patients tested negative for leishmania on the first BMA smear. All patients were treated against *leishmania* with amphotericin B (76/135, 56.3%) or antimony (77/135, 57.0%), and 13.3% (*n* = 18/135) of patients received both medications, in which amphotericin B was used as rescue treatment. The prognosis was favorable, with the exception of two deaths.

**Conclusions:**

Vigilance towards screening for leishmania infection induced HLH is imperative, particularly when there is a suspicious epidemiological history, ineffective chemotherapy, or prior to bone marrow transplantation. Early recognition, accurate diagnosis, and prompt treatment initiation can significantly alter the course of the disease and favor the prognosis in childhood with HLH secondary to VL.

## 1 Introduction

Hemophagocytic lymphohistiocytosis (HLH) is a lethal condition characterized by immunological overactivation of cytotoxic T cell natural killer cells (NK) and macrophages leading to overproduction of pro-inflammatory cytokines and injury of multiple organ systems (1). The etiology of HLH can be broadly categorized into primary (familial) HLH and secondary (sporadic) HLH. Primary HLH is caused by gene mutations and primary immunodeficiency that regulate the granulose-dependent cytotoxicity of natural killer cells and cytotoxic T lymphocytes (CTLs), including familial HLHs (FHLHs) and related immunodeficiency diseases, which mainly occur in children. Secondary HLH can be triggered in the context of various infections, definite rheumatic immune disease (macrophage activation syndrome, MAS), malignancy, and iatrogenic immune activation, affecting all age groups (2). Infection is the most common cause of secondary HLH, while *leishmania* is the most common protozoan infection-induced HLH (3).

Visceral leishmaniasis (VL), also known as Kala-azar, is caused by infection with *leishmania* and transmitted by the bite of female phlebotomine sandflies (4). It is estimated that 700,000–1 million new cases occur annually in more than 90 countries, especially in East Africa, the Mediterranean basin, Southeast Asia, and Latin America (5). According to a systematic review, a total of 150,072 VL patients have been reported in China, and 7,847 (5.2%) of them have died (6). It is primarily distributed in the northwest, with Xinjiang Uygur Autonomous Region, Gansu Province, and Sichuan Province being the top three affected areas (7). VL exhibits a broad spectrum of clinical manifestations, ranging from asymptomatic infection to persistent fever, hepatosplenomegaly, and pancytopenia, which has overlapping clinical features with HLH. It has been reported that VL-related HLH is rare in childhood, and the mortality could reach 100% without early diagnosis and treatment (8). Early recognition and treatment of VL-related HLH is critical to improving outcomes.

Recent studies have highlighted the importance of various diagnostic methods in diagnosing VL-HLH. Bone marrow aspiration (BMA) is a traditional method for diagnosing VL, but it may require multiple tests to detect the parasite in some cases (9). The rK39 rapid diagnostic test (RDT) is quick and sensitive, but it can yield false negatives in certain situations (10, 11). Serological tests, which involve detecting Leishmania antibodies or antigens, aid in auxiliary diagnosis (9). Polymerase chain reaction (PCR) and reverse transcription polymerase chain reaction (RT-PCR) molecular detection offer higher sensitivity and specificity for detecting VL-HLH, particularly when bone marrow aspiration results are uncertain (12, 13). Furthermore, when traditional methods fail, next-generation sequencing (NGS) provides a new diagnostic approach (14, 15).

VL-associated HLH is a relatively rare disease with significant diagnostic and management implications. Information on the clinical and laboratory findings and the outcome of children diagnosed with VL-associated HLH is scarce. Herein, we describe two children who were initially diagnosed with HLH but were ultimately diagnosed with VL and systematically review all reported cases of pediatric HLH secondary to VL focusing on the clinical manifestations, diagnostic methods, treatment used and outcomes, to provide evidence and reference for clinician with their early identification and treatment.

## 2 Case report

### 2.1 Case 1

**Figure 1:**
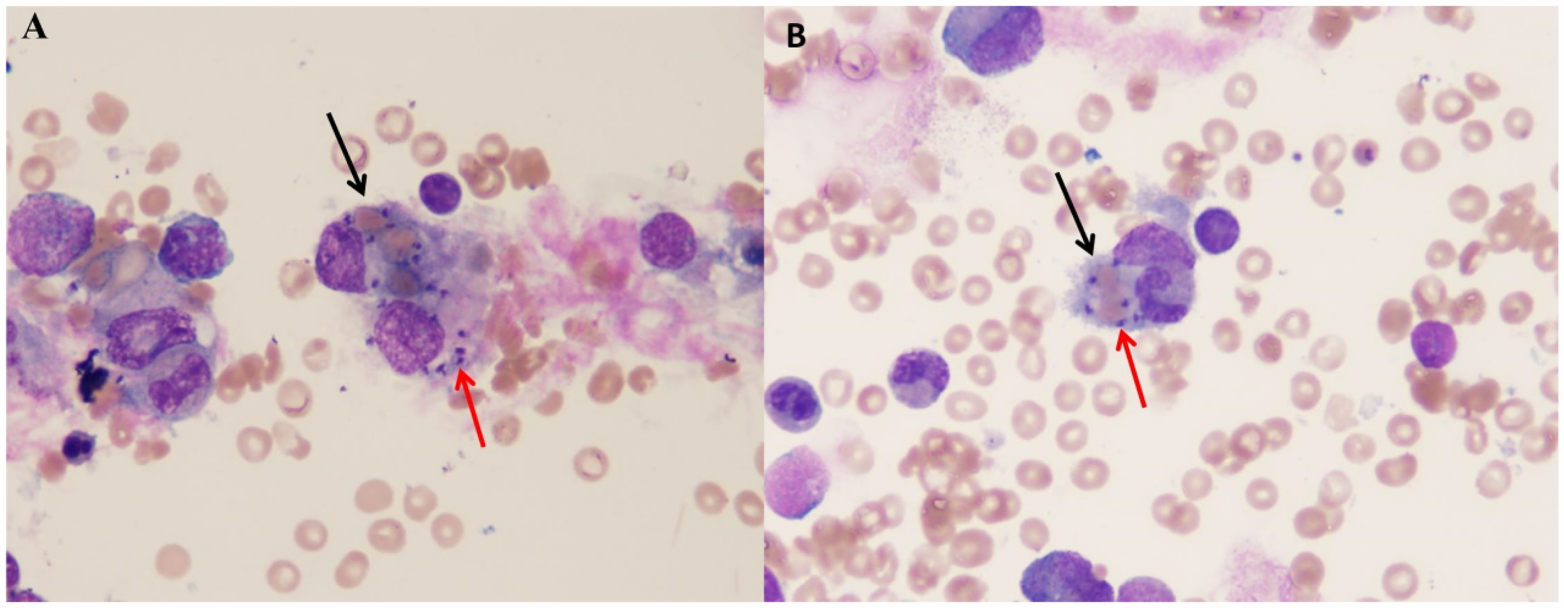
The picture showing extracellular Leishman-Donovan bodies and hemophagocytic cells in BMA [**(A)** for case 1, **(B)** for case 2]. The black arrow shows the hemophagocytic cell. The red arrow shows the Leishman-Donovan bodies.

A 15-month-old girl residing in Wen County, Gansu Province, China, was admitted to our hospital with recurrent high fever for 13 days, accompanied by cough and nausea. Her family histories were unremarkable. The patient received empirical treatment for presumed bacterial infection and platelet transfusions in the local hospital, but her symptoms were not relieved. Upon admission, physical examination revealed pallor, rales in both lungs and hepatosplenomegaly (hepatomegaly of 8 cm below the right costal margin and splenomegaly of 5 cm below the left costal margin). Hematological examinations confirmed pancytopenia, with neutropenia (0.59   ×   10^9 ^L), anemia (80 g/L) and thrombocytopenia (53   ×   10^9 ^L). The laboratory testing revealed hypertriglyceridemia (2.72 mmol/L), hypofibrinogenemia (104 mg/dl) and hyperferriinemia (>16,500 ng/ml). Serological tests for rK39, Epstein–Barrvirus (EBV), cytomegalovirus, hepatitis B, syphilis, and human immunodeficiency virus (HIV) were performed and proved to be negative (Table 1). BMA revealed signs of hemophagocytosis and Leishman-Donovan bodies (Figure 1A). Given of five out of eight diagnostic criteria for HLH, including fever, pancytopenia, hepatosplenomegaly, hypofibrinogenemia and hyperferriinemia were fulfilled (16), the leishmania amastigotes were also observed in the BMA, leading to a diagnosis of VL with secondary HLH. The patient was treated with a total of 227 mg/kg of antimony gluconate over 9 intravenous doses. Her clinical conditions dramatically improved as early as the third day of treatment. By the 14th day, fever and hepatosplenomegaly were relieved, and blood counts almost normalized. Follow-up examinations conducted over one year showed complete remission of VL without recurrence.

**Table 1:** Summary of clinical investigations.

Investigation	Case 1	Case 2
Travel history	+	+
Fever	+	+
Splenomegaly	+	+
Hepatomegaly	+	+
Cytopenias		
Hb (g/L)	80	81
Plt (109/L)	53	65
Neu (109/L)	0.59	0.68
Triglycerides (mmol/L)	2.72	2.08
Ferritin (ng/ml)	>16,500	1,437.34
Fibrinogen (g/L)	1.04	1.8
Hemophagocytosis in bone marrow	+	+
Reduction of NK7 activity	Nd	Nd
sCD25 increased	Nd	Nd
Anti-rK39 ELISA for leishmaniasis	–	–
Epstein Barr virus serology	–	–
Cytomegalovirus serology	–	–
Hepatitis B serology	–	–
Syphilis serology	–	–
HIV serology	–	–

Nd, not done.

### 2.2 Case 2

A previously 29-month-old boy was referred to us with a persistent fever and pancytopenia that had persisted for 14 days. Family history was non-contributory. Physical examination on admission revealed pallor, hepatomegaly, significant splenomegaly, and mild enlargement of cervical lymph nodes. Laboratory investigations showed low levels of hemoglobin 81 g/dl, leukocyte 1.68 × 10^9 ^L, absolute neutrophil 0.657 × 10^9 ^L, and platelet 65 × 10^9 ^L. High levels of ferritin (1,437.34 ng/ml), alanine aminotransferase (ALT) 102 U/L, AST 110 U/L and fibrinogen 1.8 g/L. Travelling to Heishui County, Sichuan Province half a month ago leads to the suspicion of VL (Table 1). Multiple hematological examinations confirmed leukopenia, anemia and thrombocytopenia. A BMA examination was performed and revealed the presence of both Leishman-Donovan bodies (Figure 1B) and hemophagocytosis. rK39 enzyme-linked immunosorbent assay (ELISA) was negative. The diagnosis of HLH secondary to VL was considered. Prompt treatment with antimony gluconate was initiated, with dosages of 0.2 g on the first day, 0.6 g on the second day, 0.3 g for the next four days, and 0.4 g for the following three days. The patient's fever, pancytopenia, and organomegaly were relieved rapidly. On the 13th day of postadmission, blood routine examination before discharge indicated improvement particularly with a decrease C-reactive protein (CRP, 1.58 mg/L) and increase in platelet 212.0 × 10^9^/L, neutrophil 2.03 × 10^9^/L, red blood cell 3.52 × 10^12^/L, hemoglobin 91.0 g/L. Abdominal examination showed the liver was around 2.5 cm subcostal, and the spleen measured about 6 cm along the I line. The patient was discharged on the 15th day and followed up for one year without any recurrence.

## 3 Systematic review of literature

### 3.1 Methods

#### 3.1.1 Data sources and searches

A systematic search was performed in MEDLINE, Web of Science, EMBASE, CNKI, VIP, CBM independently using the following terms “visceral leishmaniasis”, “black fever”, “kala-azar”, and “child”, “children”, “pediatric”, and “hemophagocytic lymphohistiocytosis”. The search was limited to articles published from January 2013 to May 2024.

#### 3.1.2 Data extraction

Two researchers independently screened the literature and extracted data. In cases where there were controversies, a third researcher was consulted until a consensus was reached. Duplicate articles were excluded during the screening process. Both abstracts and full articles were reviewed, and only articles that fulfilled the criteria of both VL and HLH were included (16, 17). Articles or cases in which primary HLH or secondary HLH was caused by other factors, including other pathogenic infections, autoimmune diseases, tumors, etc., were excluded. The extracted information from included studies included authors' names, publication year, demographic characteristics, clinical characteristics, laboratory test results, treatment, and outcomes of patients. Case reports were evaluated using the JBI criteria (18), with a total score of 8 points. The case series were evaluated using the quality evaluation tool developed by the Institute of Health Economics (IHE) in 2012, with literature meeting the requirement of 14 or more being considered high-quality (19).

#### 3.1.3 Data statistics

Descriptive analysis was performed on the small number of cases of HLH secondary to VL using frequency percentages (%), as part of the systematic evaluation. Data analysis was conducted using SPSS 23 software and Excel.

## 4 Results

### 4.1 Study selection and characteristics

**Figure 2:**
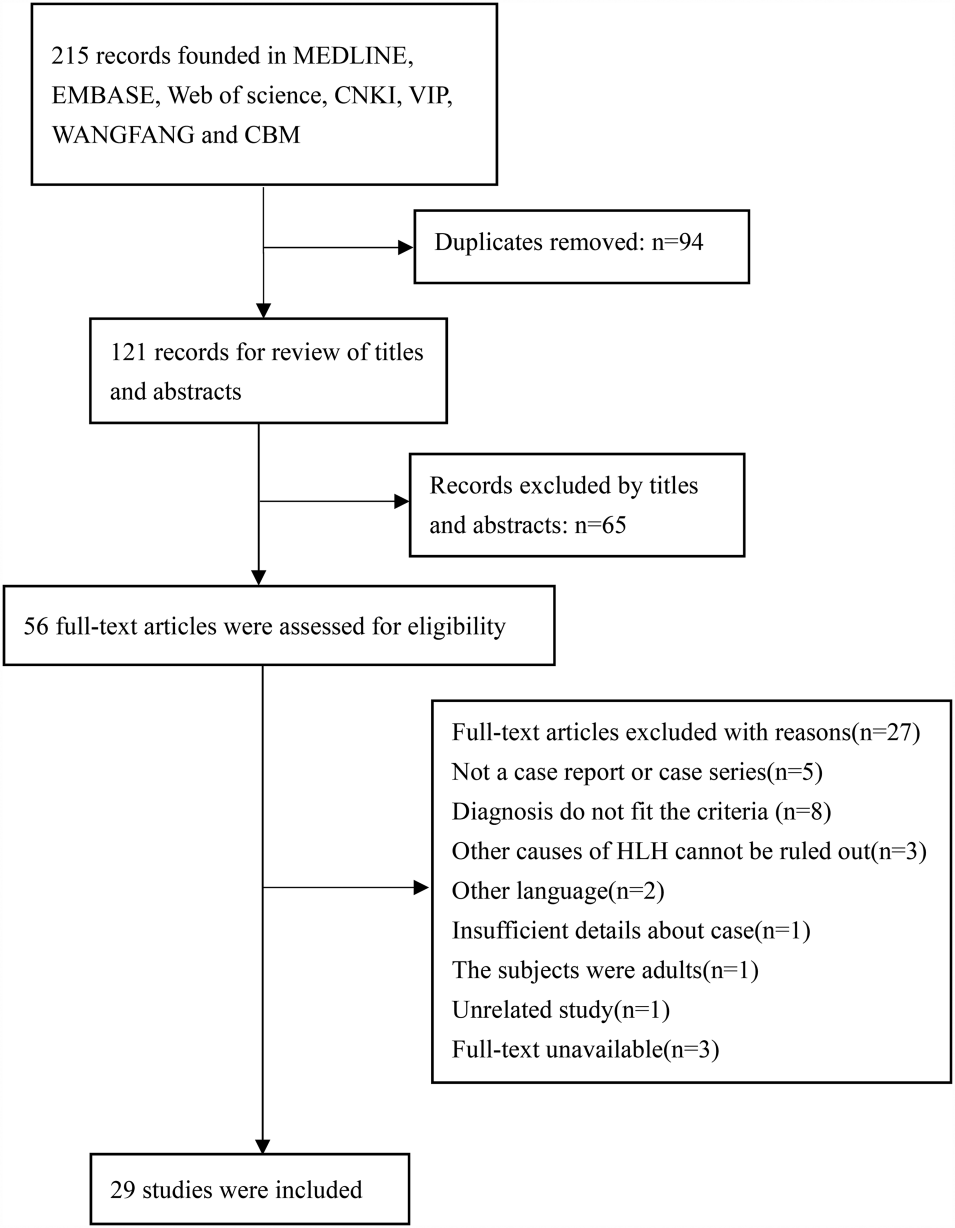
Flow diagram of study selection process.

Initial database searches identified 215 articles. After removing 94 duplicates and excluding 65 through title/abstract screening, 56 underwent full-text review. Ultimately, 29 studies (24 case reports, 5 case series) encompassing 135 cases were included (Figure 2).

The quality of included studies was assessed using JBI criteria for 24 case reports (scores: 6–8) and the IHE tool for 5 case series (scores: 10–15). Results are detailed in Table 2.

**Table 2:** The characteristics of included studies.

Author	Publication year	Type of study	Cases reported	Eligible cases	Quality evaluation (score/number of conformance)
Bolia et al. (20)	2021	Case report	1	1	6
Brum et al. (21)	2021	Case report	5	4	6
Carvalho et al. (22)	2020	Case series	39	39	13
Das et al. (23)	2015	Case report	1	1	8
Guo et al. (15)	2020	Case report	1	1	7
Yarali et al. (24)	2017	Case report	1	1	6
Bode et al. (25)	2014	Case series	13	7	13
Daher et al. (26)	2015	Case series	35	35	13
Mantadakis et al. (27)	2021	Case report	1	1	6
Singh et al. (28)	2013	Case report	1	1	7
Higel et al. (29)	2015	Case report	1	1	7
Johnson et al. (30)	2019	Case report	1	1	8
Scalzone et al. (31)	2016	Case report	1	1	7
Russo et al. (32)	2018	Case report	1	1	5
Visentin et al. (33)	2013	Case report	2	1	8
Zhang (34)	2018	Case report	1	1	7
Yi (35)	2017	Case report	1	1	7
Shi et al. (36)	2017	Case series	11	11	15
Chao et al. (37)	2021	Case report	2	2	7
Suo et al. (38)	2020	Case series	10	10	10
Dai et al. (39)	2019	Case report	1	1	6
Arora et al. (40)	2015	Case report	1	1	6
Leblanc et al. (41)	2016	Case report	1	1	6
Oudaina et al. (42)	2014	Case report	1	1	7
Melchionda et al. (43)	2014	Case report	4	1	7
Shi et al. (44)	2022	Case report	2	1	7
Li et al. (45)	2022	Case report	4	4	7
Zhang et al. (46)	2015	Case report	3	3	7
Li et al. (47)	2022	Case report	1	1	8

### 4.2 Characteristics of patients

#### 4.2.1 Demographic data

The study included 135 children with ages ranging from 3.5 months to 168 months. The male-to-female ratio was 1.3:1. Of these, 71.2% (89/125) were under 3 years old, followed by 3–6 years old (19/125, 15.2%) and over 6 years old (17/125, 13.6%). 8.9% (12/135) of the patients had positive epidemiological history, while the remaining cases were from regions where previous VL cases have been reported or VL endemic areas (Table 3). The most commonly seen countries of infection were Brazil (78/135, 57.8%) and China (36/135, 26.7%), followed by Spain (6/135, 4.4%), and Italy (3/135, 2.2%) (Tables 3, 4).

**Table 3:** Summary of demographic and clinical characteristics on presentation per case.

Author	Age (months)	Gender	Possible country of contagion	Fever	Fever duration before admission (days)	Hepatospleno-megaly	Pancyto-penia	Mode of diagnosis	Treatment to VL	outcome
Bolia et al. (20)	18	F	Nepal	+	120	+	+	rK39/BMA-smear (−)	AmB (single dose 10 mg/kg)	Cured
Brum et al. (21)	14	F	Brazil	+	NA	+	–	BMA-smear	AmB	Cured
	7	M	Brazil	+	10	+	+	rK39 + BMA-smear	AmB	Cured
	12	M	Brazil	+	90	+	+	rK39 + BMA-smear	Glucantime*8 days, nonresponse, then AmB	Cured
	24	F	Brazil	+	4	+	+	BMA-smear	AmB	Cured
Carvalho et al. (22)	5–110 (24.96)	18F/21M	Brazil	+	7–120 (23.72)	+	17/39	BMA-smear^a^ 26/37 (70.3%) + Rapid serology 32/34 (94.1%)	N-methylglucamine antimony as the first option (18/39), AmB for 8cases as rescue treatment + AmB as the first option (21/39)	Cured
Das et al. (23)	120	M	India	+	150	+	+	rK39 + BMA-smear	SSG (20 mg/kg/d, d 28)	Cured
Guo er al. (15)	9.5	F	China	+	8	+	+	mNGS (blood and sputum)/rK39 (−)/BMA-smear (−)	AmB	Cured
Yarali et al. (24)	15	M	Indian	+	10	+	+	2nd BMA-smear	AmB	Cured
Bode et al. (25)	6	F	Spain	+	18	S	+	IFT + BMA-smear	AmB (3 mg/kg/d, d 1–4 and 10)	Cured
	24	F	Amenia	+	540	S	+	IFT + PCR (blood and BMA) + BMA-culture + BMA-smear^a^	AmB (3 mg/kg/d, d 1–5, 14 and 21)	Cured
	98.4	F	Kosovo	+	21	S	+	IFT + BMA (PCR + smear)/blood PCR (−)/spleen biopsy (−)	AmB (3 mg/kg/d, d 1–5, 14 and 21)	Cured
	14.4	M	Kosovo	+	4	S	+	IFT + BMA (PCR + smear)/BMA culture (−)	AmB (2 mg/kg/d, d 1–10)	Cured
	25.2	F	Spain	+	30	S	+	IFT + BMA (PCR + smear)	AmB (3 mg/kg/d, d 1–5, 10)	Cured
	21.6	F	Spain	+	29	S	+	IFT + PCR (blood and BMA) + BMA-culture/BMA-smear (−)	AmB (2.5 mg/kg/d, d 1–10, 2nd course of AmB for VL reactivation)	Cured
	13.2	M	Spain	+	150	S	+	IFT + BMA (PCR + culture) + BMA-smear^a^	AmB (4 mg/kg/d, 3 months)	Cured
Daher et al. (26)	50.4 ± 51.6	13F/22M	Brazil	+	45 ± 50	33/35 S 21/35 H	NA	rK39 + BMA-smear^b^	Antimony (21/35), AmB for 9cases as rescue treatment + AmB (14/35)	one patient died
Mantadakis et al. (27)	84	M	Greece	+	10	+	–	BMA-smear + IFT	AmB (3 mg/kg/d, d 1–5, 14 and 21)	Cured
Singh et al. (28)	21	M	Croatia	+	28	+	+	Pre-HSCT investigations 3rd BMA-smear + BMA-PCR	AmB	Cured
Higel et al. (29)	20	M	Spain	+	30	+	+	Liver biopsy specimen-smear + BMA (PCR + smear) + IFT	AmB	Cured
Johnson et al. (30)	4	M	Spain	+	7	+	+	BMA PCR + Anti-rK39/BMA-smear (−)/skin swabs (−)	AmB (3 mg/kg/d, d 1–5, 14, 21, 28, 35 and 42)	cured
Scalzone et al. (31)	7.5	NA	Italy	+	NA	+	–	BMA-PCR + 2nd BMA-smear repeated serologic tests (−)	AmB (3 mg/kg/d, d 1–5, 7, total dose 21 mg/kg)	Cured
Russo et al. (32)	6	M	Italy	+	90	+	–	BMA-PCR + 2nd BMA-smear	AmB	Cured
Visentin et al. (33)	3.5	M	France	+	15	+	+	BMA-PCR + BMA-smear	AmB	Cured
Zhang (34)	108	M	China	+	14	+	+	rK39/BMA-smear (−)	Glucoantimony (218 mg/kg)	Cured
Yi (35)	15	M	China	+	18	+	+	BMA-smear/rK39 (−)	Glucoantimony (218 mg/kg)	Cured
Shi et al. (36)	10–96 (18)	4F/7M	China	+	12.27 ± 8.28	S	+	BMA-smear^a^ (11cases) + serology (9case)	Antimony gluconate (150–200 mg/kg)	Cured
Chao et al. (37)	24	M	China	+	12	+	–	rK39 + BMA-smear	Antimony gluconate (1.4 ml/d)	Cured
	22	F	China	+	7	S/H-	–	rK39 + BMA-smear	Antimony gluconate (1.0 ml/d)	Cured
Suo et al. (38)	22.32 ± 2.07	2F/8M	China	10/10	14 ± 7	8/10	9/10	BMA-smear^a^	Antimony gluconate (160–220 mg/kg)	Cured
Dai et al. (39)	11	F	China	+	7	+	+	rK39 + BMA-smear	Antimony gluconate (225 mg/kg)	Cured
Arora et al. (40)	168	M	Sudan	+	42	+	+	BMA-smear	AmB	Cured
Leblanc et al. (41)	21	F	Maroc	+	5	NA	+	BMA-smear + rK39/blood culture (−)	AmB (10 mg/kg/d)	Cured
Oudaina et al. (42)	36	M	NA	+	NA	+	+	IFT + BMA-smear	Antimony gluconate	Cured
Melchionda et al. (43)	5	F	Italy	+	28	+	+	rK39 + IFT + BMA PCR	AmB	Cured
Shi et al. (44)	9	F	China	+	30	+	–	rK39 + BMA-smear	SSG*21d (200 mg	Cured
Li et al. (45)	15	M	China	+	6	+	+	rK39/BMA-smear (−)	SSG*6d (180 mg/kg)	Cured
	12	F	China	+	10	S+/H−	+	BMA-smear + rK39	SSG*8d (270 mg/kg)	Cured
	5	F	China	+	12	S+/H−	+	BMA-smear + rK39	SSG*6d (200 mg/kg)	Cured
	10	M	China	+	9	S+/H−	+	BMA-smear + rK39	SSG*6d (200 mg/kg)	Cured
Peng et al. (46)	16.8	F	China	+	40	S+/H−	+	rK39 + 4th BMA-smear	SSG*6d (200–240 mg/kg)	Cured
	11	F	China	+	30	S+/H−	–	rK39 + 2nd BMA-smear	SSG*6d (200 mg/kg)	Cured
	36	F	China	+	90	+	+	rK39 + 2nd BMA-smear	SSG*6d (200 mg/kg)	Cured
Li et al. (47)	9.5	F	China	+	20	+	+	2nd BMA-smear	Antimony*3 days (240 mg/kg)	Died

M, Male; F, female; S, splenomegaly; H, hepatomegaly; NA, not available; AmB, amphotericin B; IFT, indirect immunofluorescence test; SSG, sodium stibogluconate. HSCT, Hematopoietic Stem Cell Transplantation; BMA-smear^a^, not available for times of BMA; BMA-smear^b^, not available for times and cases of BMA.

**Table 4:** Clinical features of reported patients of VL-HLH.

Characteristics	Value
Basic information	
Age	3.5–168 (*n* = 125)
≤36 months	71.2%
36–72 months	15.2%
>72 months	13.6%
Gender (male/female, *n*)	76/58 (*n* = 134)
Traveling history	8.9% (*n* = 12/135)
Fever duration before admission	4–540 (*n* = 132)
<30 days	85.4% (*n* = 76/89)
30– 60 days	4.5% (*n* = 4/89)
>60 days	10.1% (*n* = 9/89)
Symptoms [*n*/*n* (%)]	
Fever	100% (*n* = 135)
Splenomegaly	97.0% (*n* = 130/134)
Hepatomegaly	81.0% (*n* = 94/116)
Pancytopenia	69.0% (*n* = 69/100)
Others	24.4% (*n* = 33/135)
Auxiliary examination [*n*/*n* (%)]	
Hemoglobin < 90 g/L	96.9% (*n* = 1,28,132)
Platelet < 100 × 10*9/L	97.9% (*n* = 95/97)
Neutrophils < 1 × 10*9/L	77.1% (*n* = 37/48)
Triglyceride (≥3 mmol/L)	75.6% (*n* = 34/45)
Ferritine (≥500 ug/L)	72.0% (*n* = 95/132)
Fibrinogen (≤1.5 g/L)	66.7% (*n* = 24/36)
Hemophagocytosis in BMA	66.0% (*n* = 66/100)
Positive BMA	84.7% (*n* = 83/98)
Positive BMA PCRs	100% (*n* = 13/13)
Positive BMA smears	82.5% (*n* = 80/97)
Positive BMA cultures	75.0% (*n* = 3/4)
First negative BMA smears	37.8% (*n* = 14/37)
Serological positive	90.9% (*n* = 70/77)
rK39	92.7% (*n* = 51/55)
Treatment [*n n* (%)]	
AmB	56.3% (*n* = 76/135)
Antimony	57.0% (*n* = 77/135)
Antimony plus AmB	13.3% (*n* = 18/135)
HLH-04 protocol	9.6% (*n* = 13/135)
Corticosteroid	24.4% (*n* = 33/135)
Outcome [*n*/*n* (%)]	
Remission of fever	2–10 days (*n* = 18)
≤3 days	50% (*n* = 9)
4–6 days	38.9% (*n* = 7)
≥7 days	11.1% (*n* = 2)
Death [*n*/*n* (%)]	1.5% (*n* = 2/135)

#### 4.2.2 Clinical manifestations

Children included in the study presented with fever (89/89, 100%), splenomegaly (130/134, 97.0%), and hepatomegaly (94/116, 81.0%). Other symptoms were also observed in the children (33/135, 24.4%), including lymphadenopathy, edema, jaundice, and erythema (Tables 3, 4). The majority of cases had a fever duration within 30 days (*n* = 76/89, 85.4%), followed by over 60 days (9/89, 10.1%) and between 30 and 60 days (4/89, 4.5%).

#### 4.2.3 Laboratory findings

Laboratory findings at admission showed that most patients had low levels of hemoglobin (≤90 g/L, 128/132, 96.9%), platelet (≤100/L, 95/97, 97.9%), and leukocyte (≤1,000/ml, 37/48, 77.1%). Hypertriglyceridemia (34/45, 75.6%), hypofibrinogenemia (24/36, 66.7%), and hyperferritinemia (95/132, 72.0%) were found in more than half of patients. Almost all children (133 cases) were subjected to at least one bone marrow puncture, among the cases in which the number of bone marrow punctures was specified, 37.8% (*n* = 14/37) were negative for the first BMA smear. Positive evidence of VL infection found in BMA accounted for 84.7% (83/98). Patients who underwent BMA or blood PCR testing were positive in 13/13 (100%) patients, and BMA cultures were positive in 3/4 (75%) patients. More than half of bone marrow smear samples observed hemophagocytosis (66/100, 66.0%). Serological and rK39 tests were positive in 90.9% (*n* = 70/77) and 92.7% (*n* = 51/55), respectively (Table 4). Evidence of leishmania infection was detected in one child using NGS of blood and sputum due to negative results from two BMAs and serological tests (Table 3) (15).

#### 4.2.4 Treatment and outcome

Prior to confirmation of leishmaniasis infection, the included children who had received HLH-04 chemotherapy and corticosteroid therapy were 9.6% (*n* = 13/135) and 24.4% (*n* = 33/135) of patients, respectively. Additionally, one child has completed Hematopoietic Stem Cell Transplantation (HSCT), and another one is scheduled to undergo the procedure. Following diagnosis, amphotericin B (76/135, 56.3%) and antimony (77/135, 57.0%) administration were the most commonly used treatment; 13.3% (*n* = 18/135) of patients received both treatments, with amphotericin B used as rescue therapy. Regarding the duration of remission of fever, half of the patients achieved remission within 3 days of medication (9/18, 50%), one third (7/18, 38.8%) of patients within 4–6 days, one-ninth (2/18, 11.1%) of patients at 7 and 10 days after medication (23, 45). Among the patients who received treatments, more than half (10/17, 58.8%) of the patients have restored to normal within 1 month, and the remaining patients (7/17, 41.2%) have recovered for more than one month or even three months based on their clinical laboratory indexes. Regarding mortality, two patients died, one of whom likely suffered from acute kidney injury (AKI) (26), the other case involved a 9-month-old infant who was treated with antimony on the 27th day but succumbed to disseminated intravascular coagulation (DIC) three days later (47). No adverse reactions were observed in the two patients reported in our study (Table 4).

## 5 Discussion

HLH is a life-threatening immunological syndrome characterized by fever, hepatosplenomegaly, pancytopenia, hypertriglyceridemia, hypofibrinemia, hyperferritinemia, and hemophagocytosis in bone marrow aspirate (16). Primary HLH, MAS, and partial EBV-related HLH require immediate immunochemotherapy and timely HSCT for patient survival (48). Other viruses, *Mycobacterium tuberculosis*, varicella and leishmania have been reported to trigger infection associated HLH (49). VL infection which is essentially caused by *Leishmania donovani* and *Leishmania infantum*, is also an important cause of HLH because it is often not suspected (50). VL has been reported in various regions with varying prevalence, with rates of 2.1% in Germany (25), 27.5% in Brazil (26), and 41.7% in Spain (51). This variability may be attributed to regional specificity. According to our literature reports, the countries where the included children may acquire VL infection were mainly Brazil and China, which is consistent with the geographical distribution of VL reported before. HLH secondary to previously reported pediatric VL patients were mostly under 5 years old (52). For the first time, we counted 71% of the children with HLH secondary to VL were younger than three years old. This may be related to the high incidence of HLH at this age (53), or the species of leishmania infected. Previous literature suggests that children are at a higher risk of developing clinical disease as a result of *Leishmania infantum* (54). Unfortunately, most of the articles we included failed to further identify the species of leishmania.

Mortality rates of HLH in children are estimated to range from 8% to 22%, while in adults, the rate is estimated to be 40% (1). Most cases of secondary HLH can be effectively managed by controlling the underlying trigger. However, due to the extensive clinical spectrum of VL and the overlap of clinical features between VL and HLH may lead to a diagnostic delay in forms of HLH secondary to VL (55). Therefore, identifying the underlying causes of HLH post-diagnosis is crucial. Epidemiological history investigation is useful in clinical practice for recognizing the underlying causes of secondary HLH. However, a positive epidemiological history is sometimes difficult to obtain. There were only 12 (8.9%) cases with a specific VL endemic travel history in our review. The long incubation period (which can last from 2 to 6 months), endemic specificity and nonspecific clinical symptoms of VL infection may contribute to underestimated infectious agents of secondary HLH. With the growth of the economy, international trade, tourism etc., transmission of kala-azar to non-endemic areas has been reported (56), reflecting the geographic spread of this disease and the importance of considering VL in the differential diagnosis for HLH. Furthermore, investigating the travel history of the mother during pregnancy may help to identify possible fetal transmission of leishmania in young infants (27, 32). Clinicians should gather detailed epidemiological history to identify possible pediatric kala-azar infections when common causes of HLH have been ruled out.

**Figure 3:**
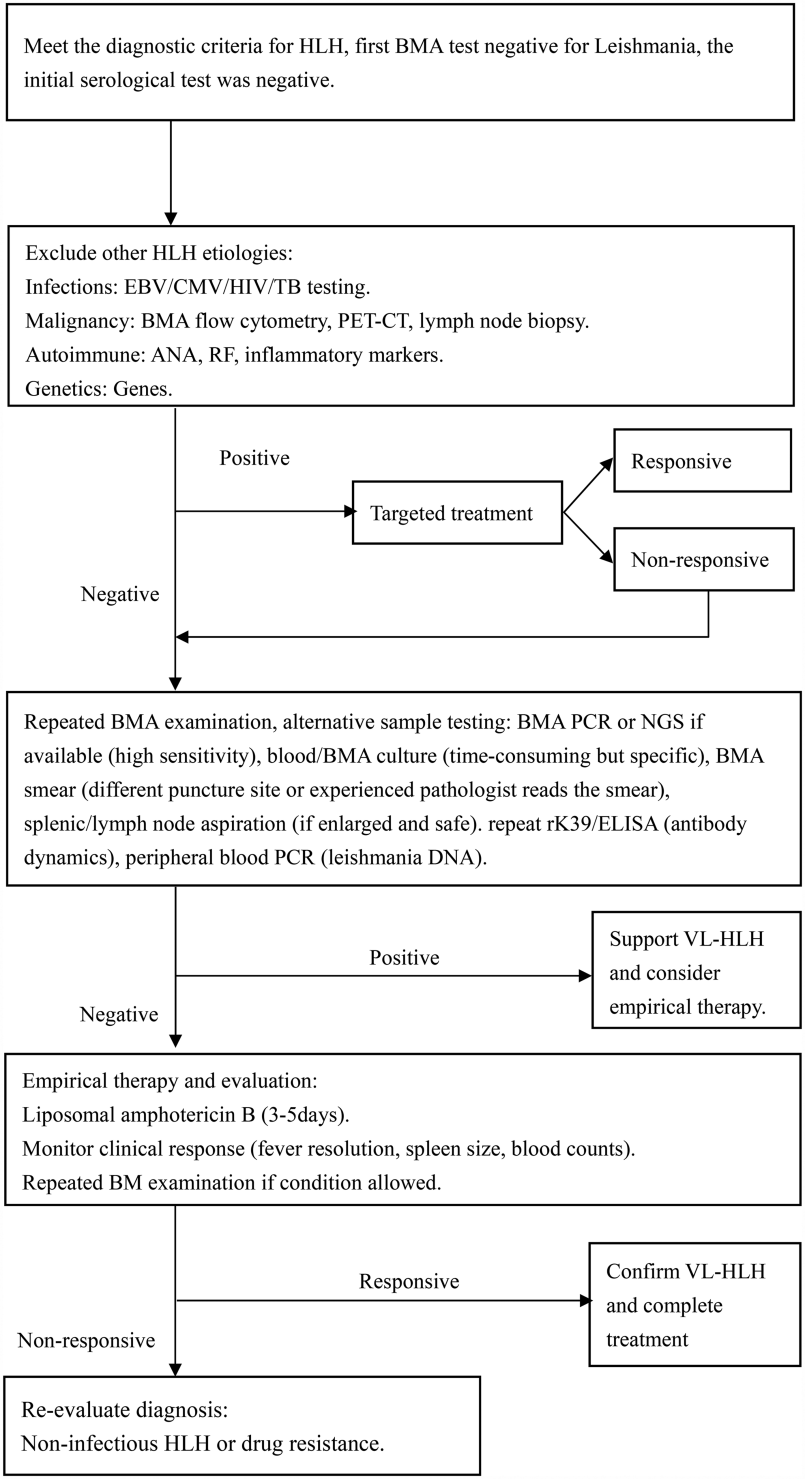
Diagnostic algorithm following an initial negative BMA examination for leishmania. CMV, cytomegalovirus; TB, tuberculosis; PET-CT, positron emission tomography—computed tomography; ANA, antinuclear antibody; RF, rheumatoid factor; DNA, deoxyribonucleic acid.

Etiological tests for HLH secondary to VL currently available include parasitological, immunological, and molecular methods. Parasitological diagnosis is the golden standard. Directly finding LD in tissues like the spleen, bone marrow, and lymph nodes could confirm VL infection. The sensitivity of tests depends on the tissue type. Splenic aspirate has a high sensitivity of up to 95% for the diagnosis of VL, but its use in clinical practice is limited due to the high risk of hemorrhage by unskilled persons (57). In our review, only one patient underwent a spleen puncture, but the result was negative (25). While lymph node sample collection is easier, its sensitivity is relatively low, ranging from 53% to 65%( 58). Moreover, our statistics indicate that lymph node enlargement is a rare manifestation among children with VL-HLH, which limits the clinical applicability of this approach. Conversely, bone marrow specimens are relatively easy to obtain and have demonstrated higher sensitivity in detecting Leishmania infection in patients with VL-HLH. The sensitivity of bone marrow smears ranges from 52% to 85% (59). The two cases we reported relied on positive BMA results to facilitate rapid diagnosis. Our study found that bone marrow smears had a positive detection rate of 82.5% for Leishmania infection in patients with VL-HLH. However, it has been reported that Leishmania amastigotes were not initially detected in BMA smears but were identified retrospectively after diagnosis (28). According to literature reports, 64% of specimens from the initial BMA test are negative (50). In our review, we analyzed the outcomes of BMAs and discovered that 37.8% of patients had negative results on their initial BMA smear. This highlights the challenges associated with detecting the Leishmania parasite in clinical samples, possibly due to the parasite's scarcity post-infection and the laboratory's testing expertise. To enhance diagnostic accuracy, repeated BMAs might be required to detect leishmania amastigotes. Parasitological culture of VL is a tedious and time-consuming procedure, which restricts its clinical application despite its high sensitivity of 97%–100%. In our review, there are only four patients underwent BMA culture, with a positivity rate of 75%. The serological tests, such as ELISA, direct agglutination test (DAT), immunofluorescence assay (IFA), and immuno-chromatographic test (ICT) is a non-invasive, rapid screening methods for VL, which are used in both endemic and non-endemic areas due to its low cost and quick results. However, its sensitivity and specificity can vary by region (60). In the cases we reported, both children's tests for rK39 antigen were negative. Statistics suggest that around 50% of patients with VL might have negative serological test results, possibly because these tests are less specific and sensitive in the early stages of the disease (27). Molecular diagnosis, including PCR techniques such as standard, nested, multiplex PCR and RT-PCR, as well as NGS plays a crucial role in detecting the parasite. In a report, the PCR-positive rate was up to 83% in serum and 100% in bone marrow aspirate samples of pediatric VL related HLH (25). It's especially useful in cases with multiple negative bone marrow smears but a strong suspicion of VL, which could reduce underdiagnosis due to limited diagnostic experience. In a systematic review of VL-related HLH cases collected before 2013, there were two cases using PCR to diagnose VL (31), while 13 cases were reported and all were positive for VL in our literature review. Although PCR for detecting VL is with high sensitivity, its use in clinical settings is limited by high costs and inadequate infrastructure in some regions. With the development of technology, the application of NGS has shown promise in detecting VL in previously intractable cases (15, 61). In summary, combining various diagnostic methods, such as multiple bone marrow smears and PCR when needed, may help in diagnosing VL at an early stage. Following a comprehensive literature evaluation, we developed a subsequent diagnostic algorithm (Figure 3) for patients fulfilling HLH diagnostic criteria with relevant travel history when initial bone marrow screening yields negative results for leishmania infection.

Correct identification of leishmania is crucial for timely treatment of children with VL-HLH. In our systematic review, 33 children received corticosteroid therapy to control excessive inflammation. However, Misuse of immunosuppressive agents may lead to the need for higher doses and a longer duration of anti-leishmanial therapy upon revised diagnosis (24, 30). Currently, VL is treated with a limited number of drugs, such as pentavalent antimony, meglumine antimoniate, injectable paromomycin, oral miltefosine, and amphotericin B (62). Amphotericin B and pentavalent antimony are the most commonly used treatments in clinical practice. In our cases, all the children included were treated with antimony or amphotericin B, and almost all children in China receive antimony due to the difficulty in obtaining amphotericin B. However, the intravenous administration of pentavalent antimony over 20–30 days is associated with significant side effects, such as cardiotoxicity, pancreatitis, and nephrotoxicity (63). The case report limitations in our study prevented us from assessing the incidence of adverse reactions to antimony. Furthermore, the rising number of reports on pentavalent antimonial resistance highlights the urgent need for more effective and safer treatments for leishmaniasis (64). In our review, 77 children were treated with antimony, and 18 experienced treatment failure or complications that resolved with remedial treatment using amphotericin B. The remaining 58 children, who were treated with amphotericin B as the first choice, were cured, except one fatality. Modified amphotericin B, which has shown efficacy and safety in regions where VL is endemic, is now recommended as a first-line anti-leishmanial drug. It offers a shorter treatment course and improved safety compared to pentavalent antimony, making it a promising alternative for treating leishmaniasis (63). Previous reports have indicated that severe HLH can lead to mortality due to multiple organ failure (65), secondary septic shock (55), hemorrhagic shock, and antimony-related myocarditis (66). In our systematic review, two deaths were reported, attributed to DIC and possible AKI. Generally, early recognition, accurate diagnosis, and prompt treatment initiation can significantly alter the course of the disease and favor the prognosis in children with HLH secondary to VL.

As an important drug for the treatment of HLH secondary to VL, the research of amphotericin B liposomal (L-AmB) has mainly focused on the optimization of dosing regimens, the efficacy of special populations and the combination therapy strategy. In a case study conducted at the First Affiliated Hospital of Xi'an Jiaotong University in China, a low-dose (0.15 mg/kg) escalating L-AmB regimen was used to achieve negative bone marrow PCR conversion with a cumulative dose of 10 mg/kg without serious adverse reactions (67). This regimen is particularly appropriate for patients with complications such as renal dysfunction or HLH. A 4.5-month-old HLH patient with H1N1 infection in Turkey had complete resolution of symptoms after treatment with L-AmB (3 mg/kg/day  ×  10 days), and there was no recurrence after 1 year of follow-up (68). In patients with HIV and HLH, L-AmB results in clinical improvement in 83% of patients with initial treatment, but it is important to note that immune reconstitution syndrome may exacerbate HLH manifestations (69). A study in Guyana, France, showed that early empiric use of L-AmB resulted in a 71% survival rate for HIV-associated HLH (69). Although L-AmB is more than 80% effective in treatment VL-HLH, the recurrence rate in immunocompromised patients is still as high as 30% (70). In this regard, some studies have recommended an extended course of therapy or a combination of immunomodulatory therapies (e.g., dexamethasone, immunoglobulin) (71).

Secondary HLH associated with VL is relatively rare and has non-specific early symptoms. Therefore, thorough medical history, and multiple and precise diagnostic tests are essential for making a timely and effective clinical diagnosis and initiating treatment. We suggest screening all children with HLH for leishmaniasis in endemic areas, particularly before starting HLH chemotherapy or HSCT. Quick remission with anti-leishmanial therapy suggests a good prognosis for children with HLH caused by VL.

## Data Availability

The original contributions presented in the study are included in the article/Supplementary Material, further inquiries can be directed to the corresponding author.
